# Clinical determinants of the severity of COVID-19: A systematic review and meta-analysis

**DOI:** 10.1371/journal.pone.0250602

**Published:** 2021-05-03

**Authors:** Xinyang Li, Xianrui Zhong, Yongbo Wang, Xiantao Zeng, Ting Luo, Qing Liu

**Affiliations:** 1 School of Stomatology, Wuhan University, Wuhan, Hubei, China; 2 Department of Computer Science, University of Illinois at Urbana-Champaign, Champaign, Illinois, United States of America; 3 Center for Evidence-Based and Translational Medicine, Zhongnan Hospital of Wuhan University, Wuhan, China; 4 Department of Epidemiology and Health Statistics, School of Health Sciences, Wuhan University, Wuhan, Hubei, China; Azienda Ospedaliero Universitaria Careggi, ITALY

## Abstract

**Objective:**

We aimed to systematically identify the possible risk factors responsible for severe cases.

**Methods:**

We searched PubMed, Embase, Web of science and Cochrane Library for epidemiological studies of confirmed COVID-19, which include information about clinical characteristics and severity of patients’ disease. We analyzed the potential associations between clinical characteristics and severe cases.

**Results:**

We identified a total of 41 eligible studies including 21060 patients with COVID-19. Severe cases were potentially associated with advanced age (Standard Mean Difference (SMD) = 1.73, 95% CI: 1.34–2.12), male gender (Odds Ratio (OR) = 1.51, 95% CI:1.33–1.71), obesity (OR = 1.89, 95% CI: 1.44–2.46), history of smoking (OR = 1.40, 95% CI:1.06–1.85), hypertension (OR = 2.42, 95% CI: 2.03–2.88), diabetes (OR = 2.40, 95% CI: 1.98–2.91), coronary heart disease (OR: 2.87, 95% CI: 2.22–3.71), chronic kidney disease (CKD) (OR = 2.97, 95% CI: 1.63–5.41), cerebrovascular disease **(**OR = 2.47, 95% CI: 1.54–3.97), chronic obstructive pulmonary disease (COPD) (OR = 2.88, 95% CI: 1.89–4.38), malignancy (OR = 2.60, 95% CI: 2.00–3.40), and chronic liver disease (OR = 1.51, 95% CI: 1.06–2.17). Acute respiratory distress syndrome (ARDS) (OR = 39.59, 95% CI: 19.99–78.41), shock (OR = 21.50, 95% CI: 10.49–44.06) and acute kidney injury (AKI) (OR = 8.84, 95% CI: 4.34–18.00) were most likely to prevent recovery. In summary, patients with severe conditions had a higher rate of comorbidities and complications than patients with non-severe conditions.

**Conclusion:**

Patients who were male, with advanced age, obesity, a history of smoking, hypertension, diabetes, malignancy, coronary heart disease, hypertension, chronic liver disease, COPD, or CKD are more likely to develop severe COVID-19 symptoms. ARDS, shock and AKI were thought to be the main hinderances to recovery.

## 1. Introduction

Coronavirus Disease 2019(COVID-19) which, was first identified in Wuhan, Hubei Province, China in December 2019, has already swept across the world. The World Health Organization (WHO) has officially declared the outbreak as a pandemic and a public health emergency [1]. As of 9^th^ March 2021, according to WHO, the number of confirmed cases around the world has surged dramatically to 116,166,652 with 2,582,528 deaths, which suggests the general mortality rate is approximately 2.22% [2]. The severity of symptoms among patients infected with COVID-19 varies considerably from being varies considerably from being asymptomatic to being a critical illness with lethal complications [3, 4].

Some researchers have suggested that there are several factors possibly responsible for the severity of COVID-19, such as hypertension, diabetes and smoking [5–7]. Therefore, we performed this systematic review and meta-analysis to explore the potential risk posed by critical medical states in COVID-19 patients. Specifically, in this study we have compared the reported clinical characteristics of patients with non-severe and severe COVID-19 in eligible published literature.

## 2. Method

### 2.1 Search strategy

The literature search was performed using international databases PubMed, Embase, Web of science and Cochrane Library using the search terms: (“COVID-19” OR “SARS-Cov-2”) AND (“characteristics” OR “clinical”): The specific search strategy is listed in S1 Table. Included studies were published between December 2019 and February 2021. The meta-analysis was conducted in compliance with the Preferred Reporting Items for Systematic Reviews and Meta-Analyses (PRISMA) statement [8].

### 2.2 Study selection

To minimize bias, two authors (LXY and ZXR) independently screened titles and abstracts and extracted potentially eligible articles. The full texts of selected articles were then carefully assessed according to the inclusion and exclusion criteria. Disagreements were resolved by discussion, with the third researcher (LQ) to reach a consensus.

### 2.3 Inclusion and exclusion criteria

Inclusion criteria:(1) case-control studies or cohort studies; (2) articles reporting the clinical characteristics and the severity of disease in patients diagnosed with COVID-19; (3) articles reporting the severity of COVID-19 and details of related factors; (4) articles reporting the specific grading standards of the severity of COVID-19.

The definition of severe disease was based on clinical symptoms, i.e., patients having severe dyspnea, extremely low oxygen saturation, respiratory distress or requiring mechanical ventilation, ICU admission or death.

The exclusion criteria: (1) review articles, letters, comments or opinions; (2) samples of less than 50; (3) incomplete information or full texts unavailable.

### 2.4 Quality assessment

All included studies were retrospective cohort studies so, the Newcastle-Ottawa Scale (NOS) was used to evaluate quality [9]. The major components include: representativeness of the exposed cohort, selection of the non- exposed cohort, ascertainment of exposure, demonstration that outcome of interest was not present at start of study, comparability, assessment of outcome, follow-up time and adequacy of follow up of cohorts. The quality rating is from 0 to 10 stars and the score≥7 stars indicates high-quality articles.

### 2.5 Data extraction

Two independent authors (LXY and ZXR) performed the data extraction by using standardized forms, which included date of publication, authors, region, number of included patients, sex, body mass index (BMI), smoking habits, comorbidities, complications, and severe or non-severe COVID-19.

### 2.6 Statistical analysis

We calculated odds ratios (ORs) in the dichotomous variables and standard mean difference (SMD) in the continuous variables with the 95% confidence intervals (CIs) and assessed publication bias using Stata 12.0. Heterogeneity was measured by I^2^ statistic. We used a Mantel-Haenszel random effects model to calculate effect sizes. In order to discover the potential sources of heterogeneity, we performed subgroup analyses and meta-regression analysis according to study location (Hubei province and outside Hubei province) and the median ages of patients. The stability of results was assessed by conducting a sensitivity analysis which omitted each study in turn. Funnel plots were used to assess for publication bias and asymmetry was taken to indicate bias. In this study, *P* values <0.05 were regarded as indicating statistical significance for the summary OR and SMD. In order to quantify the effects of risk factors on COVID-19 patients, we calculated population attributable fraction (PAF) by using the attributable fraction formula:

$$PAF=Pc\times \frac{RR-1}{RR}$$

where *Pc* is the prevalence of the risk factors in the population [10]. In this analysis, the ORs were used as approximations of RRs.

## 3 Results

### 3.1 Study selection and characteristics

**Fig 1:**
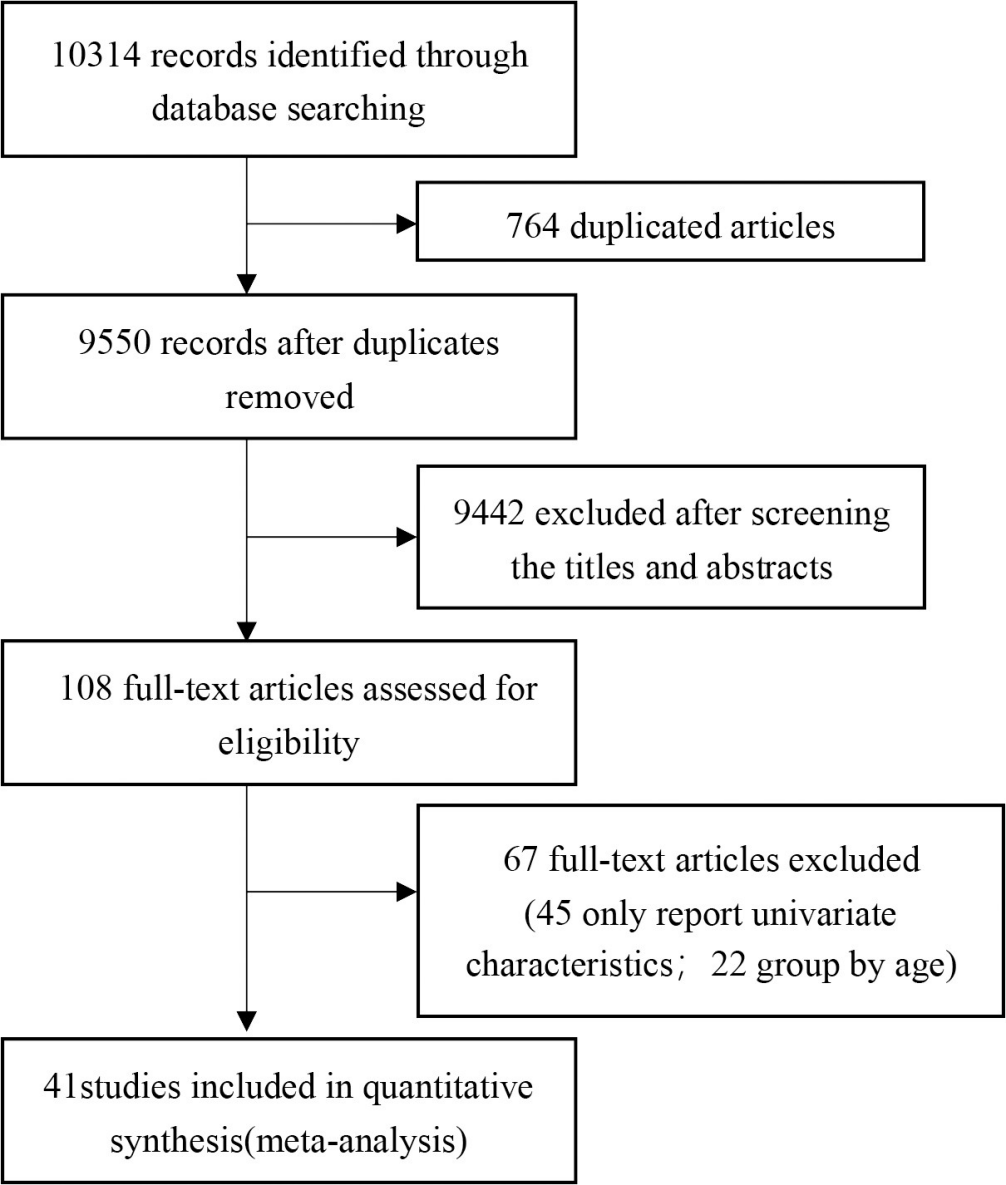
Flow diagram of literature selection.

The complete literature-search process is displayed in Fig 1. The search strategy initially retrieved 10314 articles, 10273 of which were excluded by assessing the eligibility criteria. Of these studies, 764 papers were duplicates, 9442 were perceived as unrelated research and 67 articles were in line with exclusion criteria. Finally, 41 articles [11–51] published between February 7^th^, 2020 and March 13^th^, 2021 were included, all of which were retrospective cohort studies. In total, 21060 patients were included in the meta-analysis. The characteristics of all the included studies are shown in Table 1.

**Table 1:** Characteristics of the included studies.

Author	Publication Date	Study Location	Samples(M/F)	Severe	Non-severe	Overall age (Median age/Mean age, range/SD)	Adjustments
**Wang *et al*. 2020a [11]**	Feb 7,2020	Wuhan, China	138(75/63)	36	102	56(42–68)	①,②,④,⑤,⑥,⑦,⑧,⑨,⑩,⑪,⑫,⑬,⑭,⑮,⑯
**Zhang *et al*. 2020a [12]**	Feb 19,2020	Wuhan, China	140(71/69)	58	82	57(25–87)	①,②,③,④,⑤,⑧,⑭,⑯
**Guan *et al*. 2020 [13]**	Feb 28,2020	China	1099(640/459)	173	926	47(35–58)	①,②,③,④,⑤,⑥,⑦,⑧,⑨,⑩,⑪,⑫,⑬,⑭,⑰
**Wan *et al*. 2020 [14]**	Mar 21,2020	Chongqing, China	135(73/62)	40	95	47(36–55)	①,②,④,⑤,⑧,⑨,⑩,⑫,⑬,⑮
**Hong *et al*. 2020 [15]**	Apr 24,2020	Daegu, Korea	98(38/60)	13	85	55.4(SD = 17.1)	①,②,④,⑤,⑥,⑦,⑧,⑨,⑩,⑫,⑬,⑭,⑮
**Zhang *et al*. 2020b [16]**	Apr 27,2020	Guangdong, China	1350(664/686)	229	1121	44.1(SD = 17.9)	①,②,④,⑤,⑧
**Chen *et al*. 2020 [17]**	Apr 28,2020	Zhejiang, China	145(79/66)	43	102	45.3(SD = 13.6)	①,②,③,④,⑤,⑦,⑧,⑨,⑩,⑪,⑫,⑬
**Zhao *et al*. 2020 [18]**	Apr 29,2020	Hubei, China	91(49/42)	30	61	46*	①,②,④,⑤,⑭,⑮,⑱
**Zheng *et al*. 2020 [19]**	Apr 30,2020	Hubei, China	73	30	43	43(21–76)	③,⑤
**Hu *et al*. 2020 [20]**	May 3,2020	Wuhan, China	323(166/157)	151	172	61(23–91)	①,③,④,⑤,⑥,⑦,⑧,⑨,⑩,⑪,⑫,⑬,⑭,⑲
**Cai *et al*. 2020a [21]**	May 14,2020	Shenzhen, China	383(183/200)	91	292	NA	⑲
**Wang *et al*. 2020b [22]**	May 19,2020	Guangzhou, China	275(134/141)	45	230	49(34–62)	①,②,③,④,⑤,⑥,⑦,⑧,⑩,⑪,⑫,⑬,⑰
**Buckner *et al*. 2020 [23]**	May 22,2020	Seattle, USA	99(53/46)	51	54	69(23–97)	①,②,③,④,⑤,⑦,⑧,⑨,⑲
**Yang *et al*. 2020 [24]**	May 25,2020	Wuhan, China	136(66/70)	33	103	56(44–64)	①,②,④,⑤,⑥,⑦,⑧,⑩,⑫,⑬,⑭,⑮
**Feng *et al*. 2020 [25]**	Jun 1,2020	China	476(271/205)	124	352	53(40–64)	①,③,④,⑤,⑥,⑦,⑧,⑨,⑪
**Suleyman *et al*. 2020 [26]**	Jun 16,2020	Detroit, USA	463(165/298)	141	214	57.5(16.8)	①,②,③,④,⑤,⑦,⑧,⑨,⑫,⑬,⑭,⑲,⑳
**Cao *et al*. 2020 [27]**	Jun 17,2020	Beijing, China	80(38/42)	27	53	53(SD = 20)	①,③,④,⑤,⑧,⑨
**Shahriarirad *et al*. 2020 [28]**	Jun 18,2020	Iran	113(71/42)	11	102	53.75(20–99)	①,④,⑤,⑥,⑦,⑨,⑪,⑳
**Nie *et al*. 2020 [29]**	Jun 26,2020	Henan, China	671(367/304)	72	583	44(31–53)	①,②,③,④,⑤,⑧,⑨
**Zhang *et al*. 2020c [30]**	Jul 8,2020	Zhejiang, China	771(394/337)	61	710	NA	①,③,④,⑤,⑦,⑧,⑨,⑩,⑪,⑲
**Cai *et al*. 2020b [31]**	Jul 11,2020	Shenzhen, China	298(145/153)	240	58	47(33–61)	①,②,④,⑤,⑦,⑧,⑩,⑲
**Gregoriano *et al*. 2020 [32]**	Jul 15,2020	Switzerland	99(62/37)	35	64	67(56–76)	①,②,④,⑤,⑦,⑧,⑨,⑪
**Ghweil *et al*. 2020 [33]**	Jul 17,2020	Egypt	66(48/18)	30	36	55.5(SD = 10.1)	①,②,③,④,⑤,⑧,⑲
**Yu *et al*. 2020 [34]**	Jul 17,2020	Wuhan, China	1663(838/825)	864	799	64(52–71)	①,②,③,④,⑤,⑥,⑦,⑧,⑨,⑩,⑪
**Wang *et al*. 2020c [35]**	Jul 18,2020	Wuhan, China	483(218/265)	421	62	48.4(SD = 12.4)	①,②,③,④,⑤,⑦,⑧,⑲, ⑳
**Lee *et al*.2020 [36]**	Jul 21,2020	Daegu, Korea	694(212/482)	137	557	52.1(SD = 18.29)	①,④,⑤,⑦,⑧,⑨,⑩,⑪
**Xu *et al*. 2020 [37]**	Jul 25,2020	Wuhan, China	88(36/52)	41	47	57.11(SD = 15.39)	①,④,⑤,⑦,⑧,⑨
**Wei *et al*. 2020 [38]**	Jul 29,2020	Wuhan, China	276(155/121)	14	262	51(41–58)	①,②,③,④,⑤,⑥,⑦,⑧,⑨, ⑲, ⑳
**Liu *et al*. 2020 [39]**	Aug 5,2020	Jiangsu, China	625(329/296)	64	561	44.44(SD = 17.19)	①,②,④,⑤
**Wang *et al*. 2020d [40]**	Aug 25,2020	Wuhan, China	110(48/62)	38	72	NA	①,③,④,⑤,⑥
**Ishii *et al*. 2020 [41]**	Sep 10,2020	Japan	345(198/147)	112	233	54(32–68)	①,④,⑤,⑥,⑦,⑧,⑨,⑩, ⑪
**Shu *et al*. 2020 [42]**	Sep 14,2020	Wuhan, China	293(135/158)	86	207	57.1(SD = 15.6)	①,②,④,⑤,⑥,⑧,⑪
**Du *et al*. 2020 [43]**	Sep 21,2020	Wuhan, China	164(84/80)	29	135	61.8(SD = 13.6)	①,②,③,④,⑤,⑦,⑧,⑨, ⑪,⑲
**Xiong *et al*. 2020 [44]**	Oct 22,2020	Wuhan,China	116(60/36)	55	61	58.5(47–69)	①,②,④,⑤,⑥,⑦,⑧,⑩, ⑫,⑬,⑭
**Lee *et al*. 2020b [45]**	Nov 18,2020	Korea	7339(2970/4369)	927	6412	47.1(SD = 19.0)	①,②,④,⑤,⑦,⑧,⑨,⑪
**Kim *et al*. 2020 [46]**	Nov 23,2020	Uzbekistan	843(480/363)	150	693	36(26–47)	①,②,④
**Ren *et al*. 2020 [47]**	Dec 4,2020	Wuhan, China	129(62/67)	40	89	50(34.5–61)	①,②,④,⑤,⑦,⑧,⑪
**Vial *et al*. 2020 [48]**	Dec 14.2020	Santiago, Chile	88(43/45)	18	70	49(39.5–65)	①,②,③,④,⑤,⑨
**Lv *et al*. 2021 [49]**	Jan 1,2021	Wuhan, China	409(188/321)	48	361	50.47(SD = 12.43)	①,②,④,⑤,⑧,⑨
**Zhang *et al*. 2021a [50]**	Jan 1,2021	Jining, China	78(50/28)	6	72	43.82(SD = 15.91)	①,②,③,④,⑤,⑥,⑦,⑧
**Zhang *et al*. 2021b [51]**	Feb 13,2021	Henan, China	172(92/80)	51	121	47.9(SD = 18.3)	①,②,④,⑤,⑥,⑧,⑨,⑩, ⑪,⑫,⑭,⑮

* Zhao et al. 2020 **[18]** only reported median age without age range

①:sex ②:age ③:smoking history④:hypertension⑤:diabetes⑥:cerebrovascular disease ⑦:malignancy ⑧:coronary heart disease ⑨: chronic obstructive pulmonary disease (COPD)⑩:chronic liver disease ⑪: chronic kidney disease (CKD) ⑫: shock⑬: acute respiratory distress syndrome (ARDS) ⑭: acute kidney injury (AKI) ⑮: cardiac trauma ⑯: arrhythmia ⑰: disseminated intravascular coagulation (DIC) ⑱: digestive tract injury ⑲: body mass index (BMI) ⑳: asthma

NA: not available

### 3.2 Quality evaluation

The results of the quality evaluation of the included retrospective cohort studies are displayed in Table 2. Because of the urgent need for published results on COVID-19 during the outbreak, some studies [11–15, 18, 19, 22, 23, 27, 29, 30, 34, 40, 43, 48, 49, 51] had limited follow up times.

**Table 2:** Methodological quality evaluation of enrolled studies based on Newcastle-Ottawa (NOS).

Author	Representativeness of the exposed cohort	Selection of the non- exposed cohort	Ascertainment of exposure	Demonstration that outcome of interest was not present at start of study	Comparability	Assessment of outcome	Follow-up time	Adequacy of follow- up of cohorts	Total
**Wang *et al*. 2020a [11]**	☆	☆	☆	☆	☆	☆		☆	7
**Zhang *et al*. 2020a [12]**	☆	☆	☆	☆	☆☆	☆		☆	8
**Guan *et al*. 2020 [13]**	☆	☆	☆	☆	☆	☆			6
**Wan *et al*. 2020 [14]**	☆	☆	☆	☆	☆	☆		☆	7
**Hong *et al*. 2020 [15]**	☆	☆	☆	☆	☆	☆		☆	7
**Zhang *et al*. 2020b [16]**	☆	☆	☆	☆	☆		☆	☆	7
**Chen *et al*. 2020 [17]**	☆	☆	☆	☆	☆	☆	☆	☆	8
**Zhao *et al*. 2020 [18]**	☆	☆	☆	☆	☆	☆		☆	7
**Zheng *et al*. 2020 [19]**	☆	☆	☆	☆		☆		☆	6
**Hu *et al*. 2020 [20]**	☆	☆	☆	☆	☆☆	☆	☆	☆	9
**Cai *et al*. 2020a [21]**	☆	☆	☆	☆	☆☆	☆	☆	☆	9
**Wang *et al*. 2020b [22]**	☆	☆	☆	☆	☆	☆		☆	7
**Buckner *et al*. 2020 [23]**	☆	☆	☆	☆	☆	☆		☆	7
**Yang *et al*. 2020 [24]**	☆	☆	☆	☆	☆	☆	☆	☆	8
**Feng *et al*. 2020 [25]**	☆	☆	☆	☆	☆☆	☆	☆	☆	9
**Suleyman *et al*. 2020 [26]**	☆	☆	☆	☆	☆☆	☆	☆	☆	9
**Cao *et al*. 2020 [27]**	☆	☆	☆	☆	☆	☆		☆	7
**Shahriarirad *et al*. 2020 [28]**	☆	☆	☆	☆	☆	☆	☆	☆	8
**Nie *et al*. 2020 [29]**	☆	☆	☆	☆	☆☆	☆		☆	8
**Zhang *et al*. 2020c [30]**	☆	☆	☆	☆	☆☆	☆		☆	8
**Cai *et al*. 2020b [31]**	☆	☆	☆	☆	☆☆	☆	☆	☆	9
**Gregoriano *et al*. 2020 [32]**	☆	☆	☆	☆	☆	☆	☆	☆	8
**Ghweil *et al*. 2020 [33]**	☆	☆	☆	☆	☆	☆	☆	☆	8
**Yu *et al*. 2020 [34]**	☆	☆	☆	☆	☆	☆		☆	7
**Wang *et al*. 2020c [35]**	☆	☆	☆	☆	☆		☆	☆	7
**Lee *et al*.2020 [36]**	☆	☆	☆	☆	☆	☆	☆	☆	8
**Xu *et al*. 2020 [37]**	☆	☆	☆		☆	☆	☆	☆	7
**Wei *et al*. 2020 [38]**	☆	☆	☆	☆	☆☆	☆	☆	☆	9
**Liu *et al*. 2020 [39]**	☆	☆	☆	☆	☆☆	☆	☆	☆	9
**Wang *et al*. 2020d [40]**	☆	☆	☆	☆	☆	☆		☆	7
**Ishii *et al*. 2020 [41]**	☆	☆	☆	☆	☆☆	☆	☆	☆	9
**Shu *et al*. 2020 [42]**	☆	☆	☆	☆	☆☆	☆	☆	☆	9
**Du *et al*. 2020 [43]**	☆	☆	☆	☆	☆	☆		☆	7
**Xiong *et al*. 2020 [44]**	☆	☆	☆	☆	☆	☆	☆	☆	8
**Lee *et al*. 2020b [45]**	☆	☆	☆	☆	☆☆	☆	☆		8
**Kim *et al*. 2020 [46]**	☆	☆	☆	☆	☆	☆	☆	☆	8
**Ren *et al*. 2020 [47]**	☆	☆	☆	☆	☆☆	☆	☆	☆	9
**Vial *et al*. 2020 [48]**	☆	☆	☆	☆	☆☆	☆		☆	8
**Lv *et al*. 2021 [49]**	☆	☆	☆	☆	☆☆	☆			7
**Zhang *et al*. 2021a [50]**	☆	☆	☆	☆	☆	☆	☆	☆	8
**Zhang *et al*. 2021b [51]**	☆	☆	☆	☆	☆☆	☆		☆	7

### 3.3 Summary of the meta-analysis for primary outcomes

We identified 15 risk factors in three categories (demographic characteristics, comorbidities, complications) that have impact on the severity of confirmed cases. All statistically significant factors in our analysis are summarized in Table 3.

**Table 3:** Results of the meta-analysis for primary outcomes.

Risk Factors	Number of Studies	OR (95%CI)	I^2^
Acute respiratory distress syndrome	10	39.59 (19.99–78.41)	48.7%
Shock	11	21.50 (10.49–44.06)	27.4%
Acute kidney injury	10	8.84 (4.34–18.00)	40.7%
Chronic kidney disease	17	2.97 (1.63–5.41)	68.4%
Chronic obstructive pulmonary disease	24	2.88 (1.89–4.38)	52.6%
Coronary heart disease	33	2.87 (2.22–3.71)	59.9%
Malignancy	25	2.60 (2.00–3.40)	11.9%
Cerebrovascular disease	16	2.47 (1.54–3.97)	28.4%
Hypertension	39	2.42 (2.03–2.88)	67.9%
Diabetes	39	2.40 (1.98–2.91)	55.6%
Obesity	7	1.89 (1.44–2.46)	0.0%
Advanced age	30	1.73 (1.34–2.12)	98.6%
Chronic liver disease	15	1.51 (1.06–2.17)	0.0%
Male	39	1.51 (1.33–1.71)	50.8%
Smoking history	20	1.40 (1.06–1.85)	30.6%

### 3.4 Demographic characteristics

**Fig 2:**
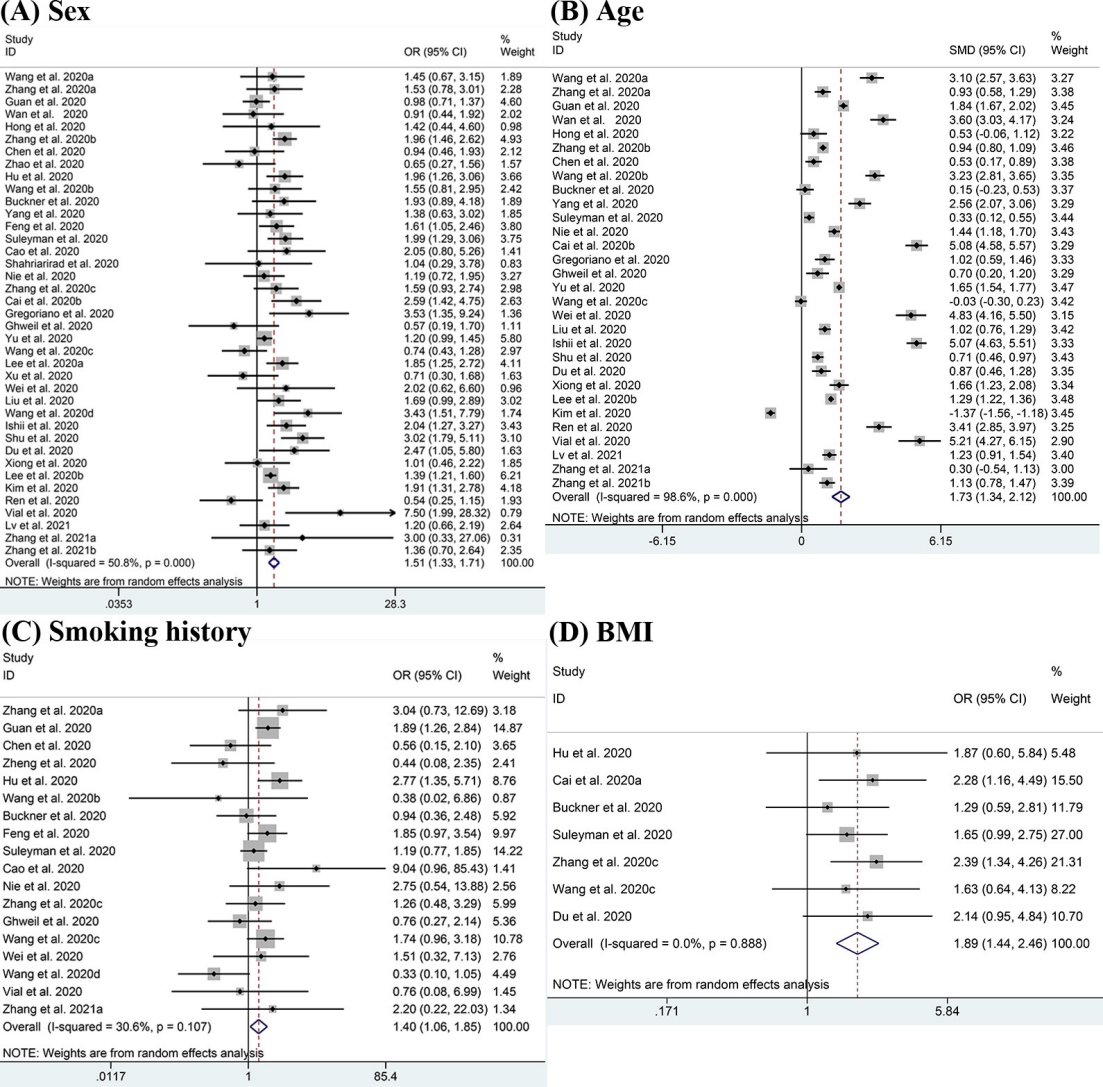
Meta-analysis of the association between demographic characteristics and severe COVID-19 disease. (A-D) Forest plots of the association between (A) sex, (B) age, (C) smoking history, (D) BMI and severe COVID-19 disease.

The demographic characteristics including sex, age, smoking history and BMI were pooled for meta-analysis and the results are displayed in Fig 2. There were 39 studies reporting on sex difference, and the pooled result showed that males were more likely to have severe disease than females (OR = 1.51, 95% CI:1.33–1.71; I^2^ = 50.8%) [11–18, 20, 22–51]. Advanced age (SMD = 1.73, 95% CI: 1.34–2.12; I^2^ = 98.6%) [11–17, 22–24, 26, 29, 31–35, 38, 39, 41–51] was also considered to be a risk factor for severe COVID-19. In all selected studies, the mean age of patients with severe conditions was older than those with non-severe conditions except for the studies from Wang 2020c [35] and Kim 2020 [46]. Patients with a history of smoking were also found to be positively associated with severe disease (OR = 1.40, 95% CI:1.06–1.85; I^2^ = 30.6%) [12, 13, 17, 19, 20, 22, 23, 25–27, 29, 30, 33, 35, 38, 40, 41, 43, 48, 50]. In studies which reported BMI, we observed higher rates of obesity (BMI≥30kg/m^2^) in patients with severe disease (OR = 1.89, 95% CI: 1.44–2.46; I^2^ = 0.0%) [20, 21, 23, 26, 30, 35].

### 3.5 Comorbidities

**Fig 3:**
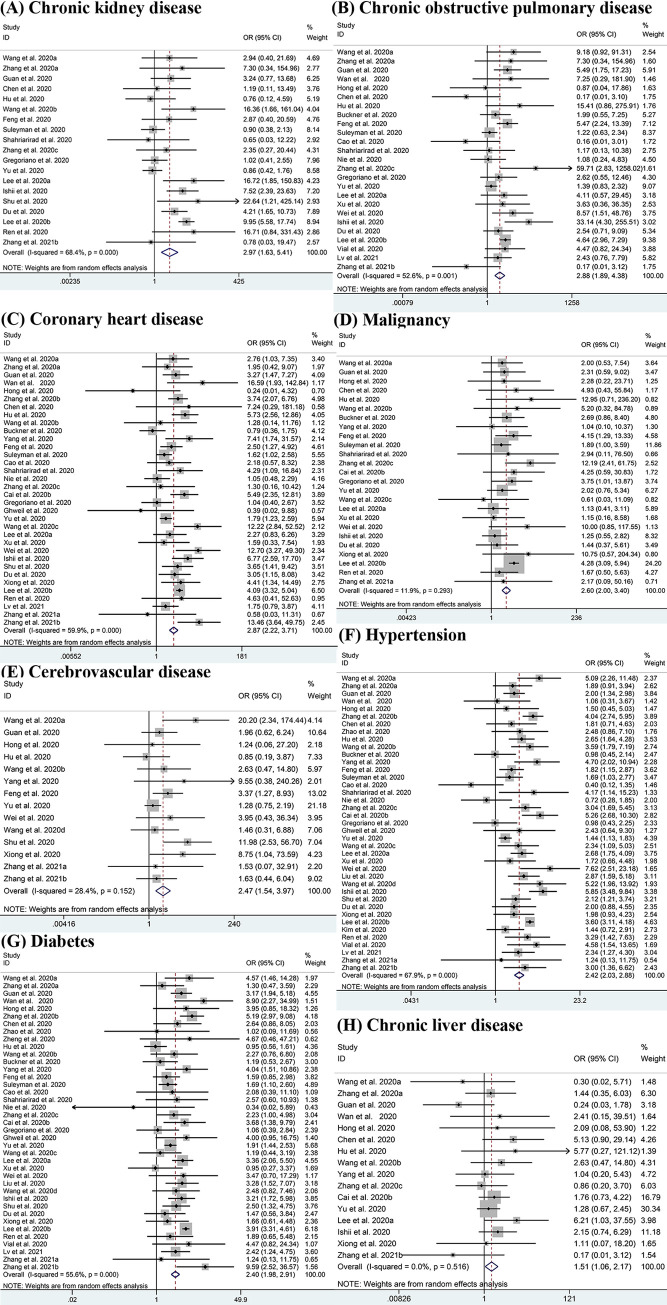
Meta-analysis of the association between comorbidities and severe COVID-19 disease. (A-H) Forest plots of the association between (A) Chronic kidney disease, (B) Chronic obstructive pulmonary disease, (C) Coronary heart disease, (D) Malignancy, (E) Cerebrovascular disease, (F) Hypertension, (G) Diabetes, (H) Chronic liver disease and severe COVID-19 disease.

Fig 3 shows the potential association between seven comorbidities and the risk of severe COVID-19 using ORs. Compared with patients having non-severe disease, there was a higher potential for patients having severe conditions to have one or more comorbidities. Results show that CKD had the highest OR value at 2.97 (95% CI: 1.63–5.41; I^2^ = 68.4%) [11–13, 17, 20, 22, 25, 26, 28, 30, 32, 34, 36, 41–43, 45, 47, 51], followed by COPD with the OR of 2.88 (95% CI: 1.89–4.38; I^2^ = 52.6%) [11–15, 17, 20, 23, 25–30, 32, 34, 36–38, 41, 43, 45, 48, 49, 51]. Other significant outcomes included coronary heart disease (OR: 2.87, 95% CI: 2.22–3.71; I^2^ = 59.9%) [11–15, 17, 20, 22–38, 41–45, 47, 49–51], malignancy (OR = 2.60, 95% CI: 2.00–3.40; I^2^ = 11.9%) [11, 13, 15, 17, 20, 22–26, 28, 30–32, 34–38, 41, 43], cerebrovascular disease (OR = 2.47, 95% CI: 1.54–3.97; I^2^ = 28.4%) [11, 13, 14, 20, 22, 24, 25, 34, 38, 40, 42, 44, 50, 51], hypertension (OR = 2.42, 95% CI: 2.03–2.88; I^2^ = 67.9%) [11–18, 20, 22–51], diabetes (OR = 2.40, 95% CI: 1.98–2.91; I^2^ = 55.6%) [11–20, 22–45, 47–51] and chronic liver disease (OR = 1.51, 95% CI: 1.06–2.17; I^2^ = 0.0%) [11–15, 17, 20, 22–26, 28, 30–32, 34–38, 41, 43–44, 47, 50]. While the analyses of asthma (OR = 1.93, 95% CI: 0.81–4.61; I^2^ = 79.4%) [26, 28, 35, 38, 45] showed no significant differences between patients with severe and non-severe disease.

### 3.6 Complications

**Fig 4:**
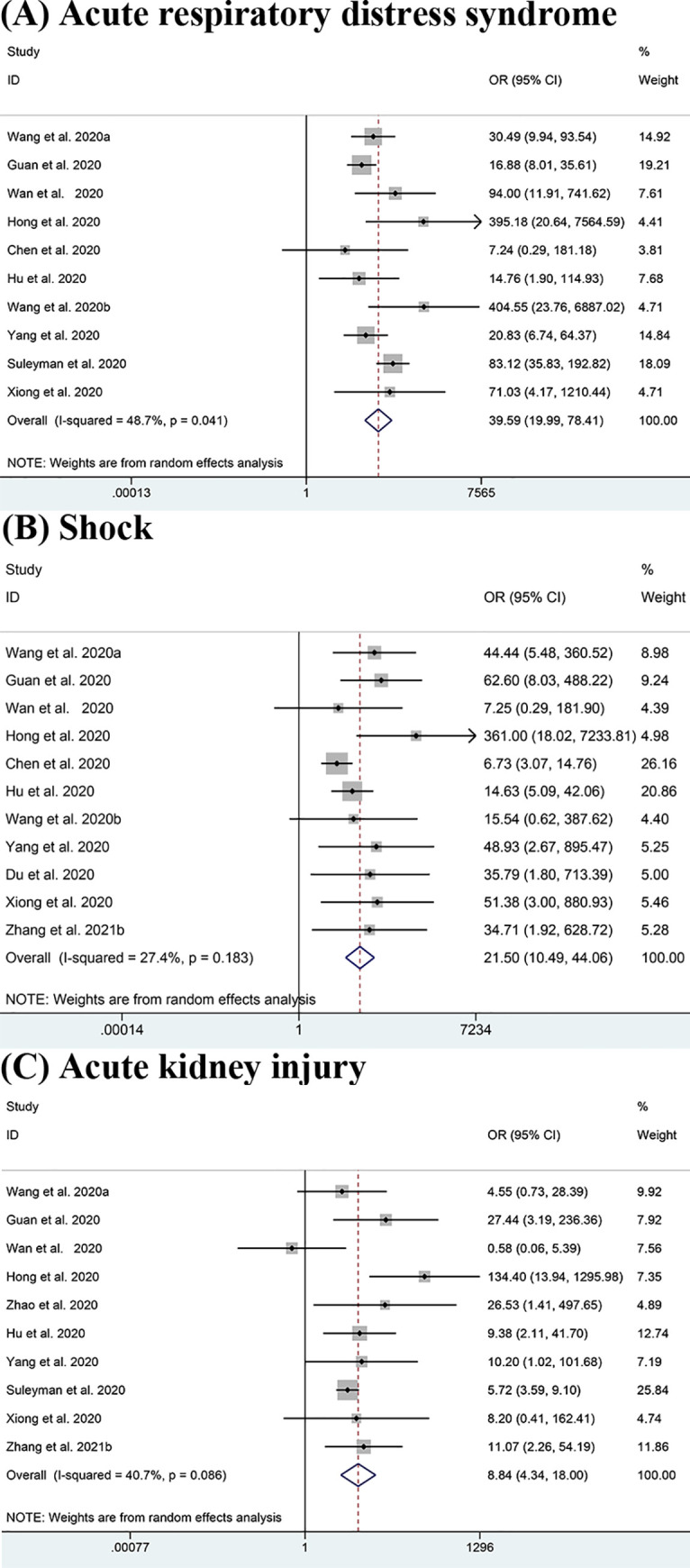
Meta-analysis of the association between complications and severe COVID-19 disease. (A-C) Forest plots of the association between (A) Acute respiratory distress syndrome (ARDS), (B) Shock, (C) Acute kidney injury (AKI) and severe COVID-19 disease.

The results shown in Fig 4 indicate that ARDS (OR = 39.59, 95% CI: 19.99–78.41; I^2^ = 48.7%) [11, 13–15, 17, 20, 22, 24, 26, 44], shock (OR = 21.50, 95% CI: 10.49–44.06; I^2^ = 27.4%) [11, 13, 15, 17, 20, 22, 24, 43, 44, 51] and AKI (OR = 8.84, 95% CI: 4.34–18.00; I^2^ = 40.7%) [11, 13–15, 18, 20, 24, 26, 44, 51] which are potential life-threatening conditions, were clearly responsible for the ICU admissions. Additionally, some other complications like DIC (OR = 29.75, 95% CI: 3.41–259.68; I^2^ = 0.0%) [13, 22], secondary infection (OR = 10.03, 95% CI: 1.99–50.55; I^2^ = 39.0%) [14, 25], arrhythmia (OR = 9.80, 95% CI:3.91–24.60; I^2^ = 0.0%) [11, 12] and cardiac trauma (OR = 7.38, 95% CI:2.28–23.82; I^2^ = 75.0%) [11, 14, 15, 18, 24, 51] also potentially increase the severity of COVID-19. Though we lacked sufficient evidence to support the results for these other complications except for shock, ARDS and AKI, the data we already had were of high consistency. However, some complications such as hypohepatia (OR = 1.53, 95% CI: 0.71–3.30; I^2^ = 0.0%) [18, 24] did not show obvious association with the severity of patients’ conditions.

### 3.7 Population attributable risk

We calculated the population attributable risk (PAR) of the population infected with COVID-19. PAR reflects the proportion of severe COVID-19 that can be attributed to exposure to a risk factor. Table 4 shows that the PARs ranged from 0.9% to 11.3%, with the estimated attributable fraction in COVID-19 patients being 11.3% for hypertension and 7.1% for obesity. It is suggested that up to 11.3% and 7.1% of severe cases could have been avoided if the prevalences of hypertension and obesity were reduced.

**Table 4:** Population attributable risks of risk factors.

Risk factor	Prevalence	OR	PAR
Smoking history	0.1351	1.40	3.9%
Obesity	0.1517	1.89	7.1%
Hypertension	0.1926	2.42	11.3%
Diabetes	0.1121	2.40	6.5%
Coronary heart disease	0.0682	2.87	4.4%
Cerebrovascular disease	0.0299	2.47	1.8%
Chronic obstructive pulmonary disease	0.0227	2.88	1.5%
Chronic liver disease	0.0278	1.51	0.9%
Chronic kidney disease	0.0155	2.97	1.0%
Malignancy	0.0256	2.60	1.6%
Shock	0.0579	21.50	5.5%
Acute respiratory distress syndrome	0.1033	39.59	10.7%
Acute kidney injury	0.0838	8.84	7.4%

### 3.8 Subgroup analysis and meta-regression analysis

**Fig 5:**
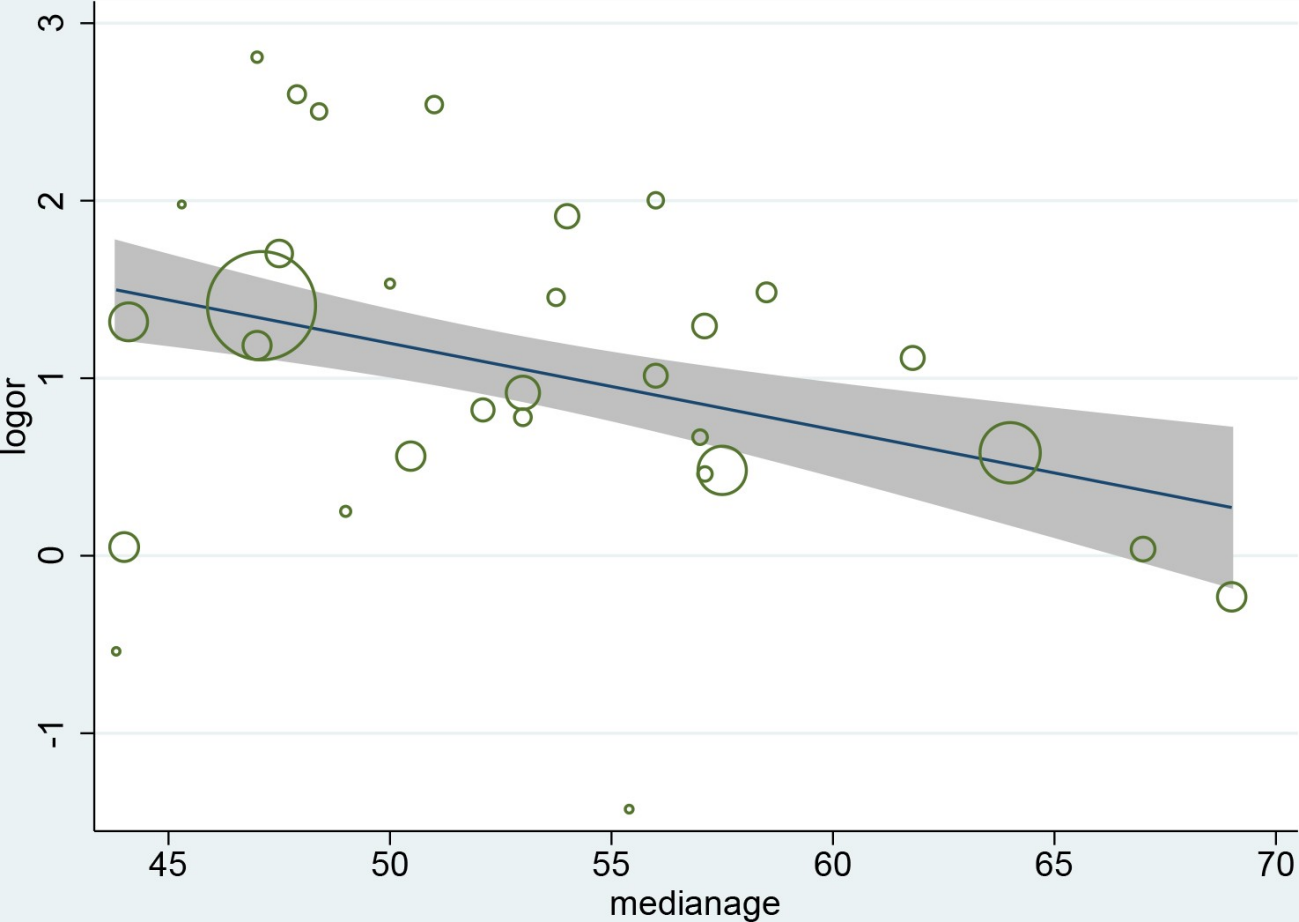
Meta-regression by median age of patients.

We performed subgroup analyses to identify the possible sources of heterogeneity of some outcomes. We tried to analyze several factors such as study location, publication date, sample size and patients’ average age of which study location gave a relatively meaningful result as shown in Table 5. This shows high consistency among the studies conducted in Hubei province but the heterogeneity of the studies outside Hubei province is also significant. Interestingly, the studies conducted in Hubei province show the ORs of ARDS was much lower than other regions. The meta-regression analysis demonstrates that the association between coronary heart disease and increased severity was influenced by age (P = 0.013), and the discrepancy between patients’ median ages in eligible studies partly contributed to the heterogeneity. (Fig 5)

**Table 5:** Subgroup analysis according to study location.

	Hubei Province				Outside Hubei Province			
	N	OR (95%CI)	I^2^	P	N	OR (95%CI)	I^2^	P
**COPD**	8	2.78(1.53,5.06)	25.7%	0.22	17	2.67(1.53,4.68)	58.9%	0.001
**CKD**	6	3.82(1.44,8.11)	13.5%	0.33	10	3.64(1.55,8.58)	69.1%	0.001
**ARDS**	5	25.25(12.32,51.74)	0.0%	0.80	6	61.22(19.03,196.94)	67.8%	0.008
**AKI**	5	8.65(3.42,21.85)	0.0%	0.90	5	9.77(2.65,36.01)	71.1%	0.08

### 3.9 Sensitivity analysis and publication bias

By excluding every article in turn, we conducted a sensitivity analysis and there was no evident change in either the significant outcomes or in heterogeneity. To assess the risk of publication bias, we drew a funnel plot for each factor. The results, displayed in S1 Fig, show that nearly all the funnel plots are symmetrical except for cerebrovascular disease, which suggests the risk of publication bias is low in our analysis.

## 4 Discussion

This study has examined the potential risk factors of severe COVID-19 such as demographic characteristics, comorbidities and complications to assist clinicians with allocation of medical resources. The higher the ORs of the risk factors, the greater the risks they bring. Our analysis has shown that males, obese patients (BMI ≥ 30kg/m^2^), patients with advanced age or a smoking history faced a greater risk of having severe COVID-19. This finding has also been supported by some previous studies [52–54]. However, some of the results of one meta-analysis were not consistent with our analysis, since they found gender irrelevant to the severity of COVID-19, which may possibly be due to the relatively small sample size [55]. The comorbidities including hypertension, diabetes, CKD, coronary heart disease, COPD, cerebrovascular disease and chronic liver disease could probably aggravate the illness and this outcome was consistent with other studies. [5, 7, 55–57] Complications including shock, AKI and ARDS were the main obstacles to recovery. Therefore, more attention should be paid to those obese elderly males with a history of smoking, and the above comorbidities or complications since they are more likely to develop the severe from of the disease. Although four previous studies [52, 53, 55, 57–59] have reported some risk factors for severe COVID-19, our analysis involved and reported on new factors obesity and chronic liver disease. In this study, the quality of evidence was higher since all the included studies were cohort studies rather than case-control studies, and we have included 41 eligible studies that were published from February,2020 to March, 2021, spanning a period of seven months.

Some studies have suggested that innate immunity and some factors associated with sex chromosomes may lead to the differences in susceptibility and inflammation between females and males. For instance, the X chromosome in the female has encoded some immune regulatory genes which caused lower viral load levels. TLR7, a Toll-like kind of receptor, which is higher in females than males, could enhance immune responses and boost the resistance to COVID-19 [60]. Besides, the circulating concentration of ACE2 (a functional cellular receptor of SARS-CoV-2) is higher in males than females, which could increase the susceptibility to SARS-CoV-2 [61]. Males also smoked at a higher rate than females [62] and that could result in a more vulnerable respiratory system. Therefore, it seems reasonable that women have been found to be more resistant to COVID-19. As for smoking, it suppresses the antiviral mechanisms and alters some cytokine patterns which play a role in the innate mucosal immunity [63]; the viral replication and the severity of COVID-19 would increase to some extent as a result. One study shows that smoking can also enhance the expression of ACE2 [64] and this would raise the susceptibility. With regard to age, it was evident that elderly people had a higher prevalence of comorbidities like diabetes [65] and complications like shock [66], but the OR was still significant when controlling for the above confounding factors. The natural decrease in functional reserve brought about by the physiological aging process could reduce elders’ capability to resist infections like COVID-19. Based on the analysis of an increasing number of studies reporting details of BMI, we considered obesity as a risk factor. Obesity could cause more endothelial dysfunction [67] and weaken the immunocompetent cells especially their cytotoxic cell responses [68]. The high level of ACE2 receptor expression in adipocytes may turn the adipose tissue into a viral carrier which could spread SARS-CoV-2 to other organs.

We also found some comorbidities were potentially high-risk factors for severe disease and this is consistent with other recent studies [53, 55, 57–59]. For example, as a chronic respiratory illness, COPD has already led to airflow blockage and so raises the risk of respiratory complications like ARDS. Psychologically, when patients with COPD get COVID-19, they seem to easily become very afraid and anxious [69]. Negative feelings may drive misbehavior and go against recovery, so psychological support is considered important. Though the degree varied, a large number of COVID-19 patients had hypoxaemia which may cause injury or apoptosis of cardiomyocytes [70]. As a result, patients with coronary heart disease were more likely to suffer from severer conditions like heart failure which can easily lead to death. For patients with hypertension, some kinds of blood pressure medications can enhance the expression of ACE2 and leave patients more susceptible to COVID-19 symptoms [71, 72]. However some other studies have shown that ACE2 plays an anti-inflammatory role in RAS and protect patients from ARDS [73, 74]. Therefore, the incidence of COVID-19 was lower but the severity was higher in patients with hypertension. Additionally, prolonged hypertension damages the vascular structure leaving the patients susceptible to serious infections. In terms of diabetes, this could compromise and weaken patients’ immune system thus worsening their conditions [75]. The dysfunctional pro-inflammatory cytokine responses due to diabetes could also increase the severity of COVID-19 [7]. Whether malignancy does affect the state of illness or lead to a poor prognosis is still controversial. Though our analysis showed there was a potential relationship between malignancy and severe condition, some articles had conflicting outcomes, and a few articles have even suggested that some patients with cancer may have better clinical outcomes since the possible emergence of cytokine storm was dampened because of their compromised immune systems [76, 77]. In addition to the above results, we have also found chronic renal disease (OR = 2.97, 95% CI: 1.63–5.41) was a potential risk factor for severe COVID-19, although a previous analysis had showed that it was not significantly associated with severe conditions [52]. It is likely that patients with CKD suffer from dysregulation of the immune system [78], which possibly contributed to the increase in severity. A study with similar results to ours has also reported a potential link between chronic liver disease and severe COVID-19 [79]. The hepatic insufficiency of COVID-19 patients could be worsened by potentially hepatotoxic drugs such as remdesivir, lopinavir and ritonavir used to treat the disease, causing more severe conditions [80]. More analyses need to be done in order to provide sufficient information and draw accurate conclusions about the controversial results above.

According to our analysis, ARDS, shock and AKI were the three major complications playing a role in severe conditions. Due to impaired gas exchange and serious inflammation in the alveolar space, patients with ARDS had significant need of ventilation [81]. Some studies [82, 83] suggested tissue plasminogen activator (tPA) may be useful in saving such patients’ lives in the face of lack of mechanic ventilation in some areas. AKI is prevalent among severe patients and can be fatal especially when patients required renal replacement therapy [84]. Similarly, shocked patients can die without timely treatment resulting from the lack of effective circulating blood volume. Therefore, it is essential to prevent the occurrence of the above complications and give prompt first aid treatment when such complications occur.

We believe the PAFs we calculated are meaningful for public health, because the risk of severe cases is likely to decrease in the wake of reduction in the prevalence of certain conditions. In our analysis, the top risk factor was hypertension with the score of 11.3%, indicating that though the OR of hypertension was not relatively high among all the factors, it did play a more important role than we had thought. The PAFs of ARDS (10.7%) and AKI (7.4%) were also high which showed that they possibly make a considerable contribution to the severity of COVID-19, but there are no specific clinical guidelines to prevent them. Therefore, more attention should be given to some other factors like obesity (7.1%) and diabetes (6.5%) since they can to some extent be prevented through practical guidelines [85] and public health campaigns. The other factors ranged from 0.9% to 5.5% and half of them were around 1.0%, suggesting that they exert less effects on the severity of COVID-19.

We should acknowledge that there are some limitations which may affect the accuracy of the outcomes and should be considered. First, some studies did not explicitly show their criteria for severe patients and non-severe patients, meaning the actual severity of patients with these two outcomes may not be consistent with those studies. Second, detecting significant heterogeneity in some analyses, we chose to use a random effects model to process data but did not identify the clear sources of heterogeneity through subgroup analyses. Third, the majority of the patients came from China, so they may not be representative of all patients across the world and so results should be carefully evaluated and accepted cautiously. The conclusion can be updated when more studies from outside China are published. Forth, we have tried to include studies from different cites but some articles may still include duplicated cases since they seldom mentioned the detailed composition of patient groups.

## 5 Conclusion

In summary, patients with COVID-19 who have the following characteristics: male gender, advanced age, a history of smoking, obesity, hypertension, diabetes, malignancy, coronary heart disease, hypertension, COPD, CKD and chronic liver disease, were more likely to develop the severe form of the disease. The emergence of complications like shock, ARDS, AKI generally increased the risk of the disease developing into the severe condition.

## Supporting information

S1 Checklist PRISMA 2009 checklist. (DOC)

S1 Fig Funnel plot of (A) sex, (B) smoking history, (C)age, (D) BMI, (E) Chronic kidney disease, (F) Chronic obstructive pulmonary disease, (G) Coronary heart disease, (H) Malignancy, (I) Cerebrovascular disease, (J) Hypertension, (K)Diabetes, (L) Chronic liver disease, (M) Acute respiratory distress syndrome (ARDS), (N) Shock, (O) Acute kidney injury (AKI) and severe COVID-19 disease for the assessment of publication bias.
(TIF)

S1 Table Search strategy in four electronic databases. (DOCX)

## Abbreviations

COVID-19: Coronavirus Disease 2019
WHO: World Health Organization
OR: odds ratio
SMD: standard mean difference
COPD: Chronic obstructive pulmonary disease
CKD: Chronic kidney disease
AKI: acute kidney injury
ARDS: acute respiratory distress syndrome
CI: confidence interval
