# Supplementary figures and images for: Clinical determinants of the severity of COVID-19: A systematic review and meta-analysis
Source: PLoS One. 2021 May 3;16(5):e0250602. doi: 10.1371/journal.pone.0250602 (PMC8092779; doi:10.1371/journal.pone.0250602)

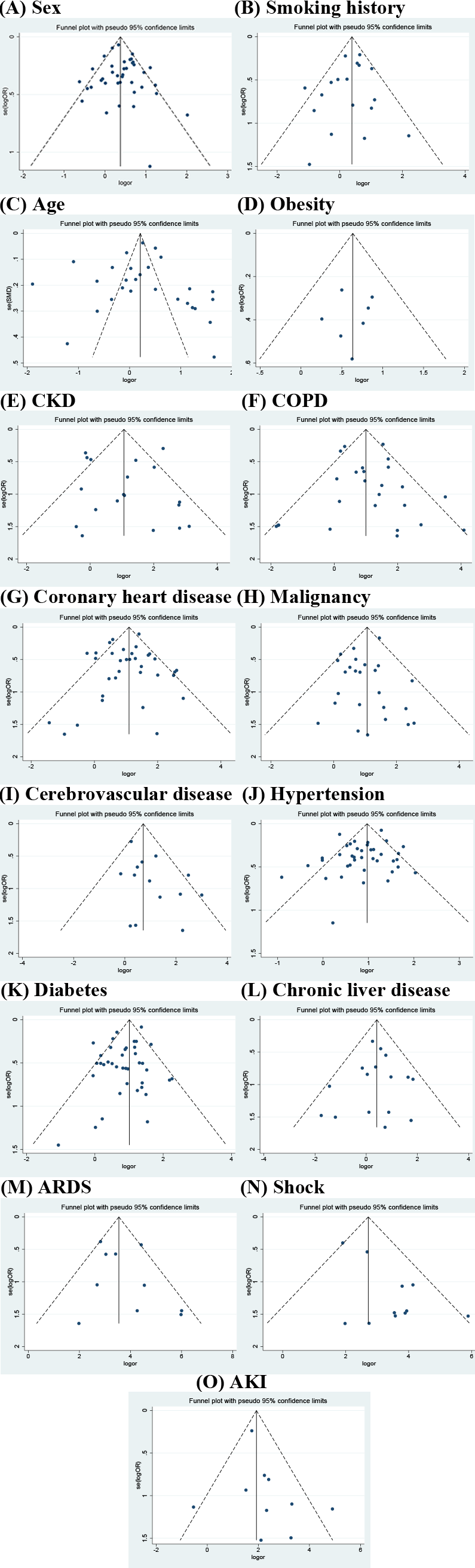

Supplement: S1 Fig — Funnel plot of (A) sex, (B) smoking history, (C)age, (D) BMI, (E) Chronic kidney disease, (F) Chronic obstructive pulmonary disease, (G) Coronary heart disease, (H) Malignancy, (I) Cerebrovascular disease, (J) Hypertension, (K)Diabetes, (L) Chronic liver disease, (M) Acute respiratory distress syndrome (ARDS), (N) Shock, (O) Acute kidney injury (AKI) and severe COVID-19 disease for the assessment of publication bias. (TIF) [file pone.0250602.s002.tif]
