# Supplementary material for: Clinical determinants of the severity of COVID-19: A systematic review and meta-analysis
Source: PLoS One. 2021 May 3;16(5):e0250602. doi: 10.1371/journal.pone.0250602 (PMC8092779; doi:10.1371/journal.pone.0250602)
Supplement: S1 Table — (DOCX) [file pone.0250602.s003.docx]

S1 Table. Search strategy in four electronic databases

| Database | Search strategy |
| --- | --- |
| Pubmed | #1 COVID-19 [Title/Abstract] OR SARS-CoV-2 [Title/Abstract]  #2 characteristics [Title/Abstract] OR clinical [Tile/Abstract]  #3 hypertension [Title/Abstract] OR diabetes [Title/Abstract] OR COPD [Title/Abstract] OR chronic obstructive pulmonary disease [Title/Abstract] OR obesity [Title/Abstract] OR smoking [Title /Abstract] OR coronary heart disease [Title/Abstract] OR malignancy [Title/Abstract] OR chronic kidney disease [Title/Abstract] OR [chronic](javascript:;) [liver](javascript:;) [disease](javascript:;) [Title/Abstract]  (#1 AND #2 OR #3) |
| Embase | #1 'COVID-19': ti,ab,kw OR 'SARS-CoV-2': ti,ab,kw  #2 'characteristics: ti,ab,kw OR 'clinical' :ti,ab,kw  #3 'hypertension': ti,ab,kw OR 'diabetes': ti,ab,kw OR 'COPD': ti,ab,kw OR 'chronic obstructive pulmonary disease': ti,ab,kw OR 'obesity': ti,ab,kw OR 'smoking': ti,ab,kw OR 'coronary heart disease': ti,ab,kw OR 'malignancy': ti,ab,kw OR 'chronic kidney disease': ti,ab,kw OR '[chronic](javascript:;) [liver](javascript:;) [disease](javascript:;)': ti,ab,kw  #1 AND #2 OR #3 |
| Web of Science | TS= (COVID-19 OR SARS-CoV-2) AND TS= (characteristics OR clinical OR hypertension OR diabetes OR COPD OR chronic obstructive pulmonary disease OR obesity OR smoking OR coronary heart disease OR malignancy OR chronic kidney disease OR [chronic](javascript:;) [liver](javascript:;) [disease](javascript:;)) |
| Cochrane of Library | #1 ' COVID-19' OR 'SARS-CoV-2'  #2 'characteristics' OR 'clinical'  #3 'hypertension' OR 'diabetes' OR 'COPD' OR 'chronic obstructive pulmonary disease' OR 'obesity' OR 'smoking' OR 'coronary heart disease' OR 'malignancy' OR 'chronic kidney disease' OR '[chronic](javascript:;) [liver](javascript:;) [disease](javascript:;)'  #1 AND #2 OR #3 |
